# Therapeutics for Inflammatory-Related Diseases Based on Plasmon-Activated Water: A Review

**DOI:** 10.3390/ijms19061589

**Published:** 2018-05-28

**Authors:** Chih-Ping Yang, Yu-Chuan Liu

**Affiliations:** Department of Biochemistry and Molecular Cell Biology, School of Medicine, College of Medicine, Taipei Medical University, 250 Wuxing St., Taipei 11031, Taiwan; d119099007@tmu.edu.tw

**Keywords:** plasmon-activated water, gold nanoparticles, anti-inflammatory, medicine, animal disease model

## Abstract

It is recognized that the properties of liquid water can be markedly different from those of bulk one when it is in contact with hydrophobic surfaces or is confined in nano-environments. Because our knowledge regarding water structure on the molecular level of dynamic equilibrium within a picosecond time scale is far from completeness all of water’s conventionally known properties are based on inert “bulk liquid water” with a tetrahedral hydrogen-bonded structure. Actually, the strength of water’s hydrogen bonds (HBs) decides its properties and activities. In this review, an innovative idea on preparation of metastable plasmon-activated water (PAW) with intrinsically reduced HBs, by letting deionized (DI) water flow through gold-supported nanoparticles (AuNPs) under resonant illumination at room temperature, is reported. Compared to DI water, the created stable PAW can scavenge free hydroxyl and 2,2-diphenyl-1-picrylhydrazyl radicals and effectively reduce NO release from lipopolysaccharide-induced inflammatory cells. Moreover, PAW can dramatically induce a major antioxidative *Nrf2* gene in human gingival fibroblasts. This further confirms its cellular antioxidative and anti-inflammatory properties. In addition, innovatively therapeutic strategy of daily drinking PAW on inflammatory-related diseases based on animal disease models is demonstrated, examples being chronic kidney disease (CKD), chronic sleep deprivation (CSD), and lung cancer.

## 1. Introduction

Since gold nanoparticles (AuNPs) possess well-defined localized surface plasmon resonance (LSPR) bands in the UV–near infrared (IR) regions, they are commonly used in surface-enhanced Raman scattering (SERS) for improved diagnostic imaging [1,2,3] and in photothermal ablation of tumors [4,5,6]. Recently, AuNPs have been widely utilized in innovatively therapeutic strategies on various cancers [7,8,9]. Saha et al. reported that gold nanoparticle reprograms pancreatic tumor microenvironment and inhibits tumor growth [10]. Melamed et al. reported that AuNPs can disrupt the tumor microenvironment, thus, serve as potential mediators of microenvironment-targeted therapy [11]. Ali et al. reported that nuclear membrane-targeted AuNPs inhibit cancer cell migration and invasion [12].

Hydrogen can act as a therapeutic antioxidant by selectively reducing cytotoxic oxygen radicals. As reported by Ohsawa et al. [13], an acute rat model was used, in which damage of oxidative stress was induced in the brain via focal ischemia and reperfusion. Experimental results indicated that inhalation of hydrogen can significantly suppress brain injury of rats. Therefore, hydrogen may be served as an effective anti-oxidant therapy. Hydrogen can reach and react with cytotoxic reactive oxygen species (ROS) due to its ability of rapid diffusion across membranes. Thus, it markedly protects against oxidative damage. In treatment, dissolved hydrogen in saline could easily be delivered intravascularly. In prevention, saturated hydrogen in water could be readily controlled. Notably, H_2_ has no risk of flammability or explosion at a concentration of less than 4.7% in air, as commented by the authors. Since then, hydrogen-rich water has been widely used as alternatively therapeutic topics on many medical diseases [14,15,16,17,18]. Moreover, acidic cosmetic water acts against *Staphylococcus aureus*, has anti-inflammatory properties, and is a superoxide anion radical scavenger [19]. Also, sulfurous water has an antioxidative effect on protection against lipid and protein oxidation [20]. The free radicals were significantly inhibited by natural water with sulfhydryl concentrations ranging from 16 to 1 μg mL^−1^ for hydroxyl radicals [21]. However, these kinds of water are not pure ones. Their special functions are attributed to additional additives, such as hydrogen gas and minerals. The water itself is still regarded as an inert solvent.

It is well known that liquid water plays a central role in various chemical processes owing to its defects deviated from a perfect tetrahedral symmetry structure of a hydrogen-bond (HB) network. In addition, the HB network of liquid water is flexible dynamic, in which hydrogen bonds are broken and reformed at equilibrium on picoseconds. This makes the corresponding examination on its instantaneous structure challenging [22,23,24]. Because our full understanding on water in its molecular-level structure is not enough, so far all the reported physical and chemical properties of water are built on bulk water [23]. However, these conventional properties of bulk water can be changed by the effects of temperature [25,26], solutes [27,28,29,30], external fields, and the environment that is associated with the strength of HBs [31]. Actually, the HB network is abruptly interrupted on the surface or interface while it contacts with a heterophase [32,33,34,35,36]. In addition, water confined into nanoscale materials also expresses different physical properties [37,38,39,40,41]. Compared to bulk water, the unique properties of water are present in the confined environment or in the interfacial phase in which the present other species are necessary for keeping its unique properties. This makes the inherent properties of pure liquid water with reduced HB can’t be independently exhibited.

Engineered water prepared from electro-spraying water vapor owns reactive oxygen species (ROS), which can inactivate foodborne microorganisms, as shown in the literature [42]. In addition, it can be applied in the food industry served as clean alternatives to conventional disinfection ways [43,44]. It is known that HBs of water would be destroyed at raised temperatures or adding salt ions in it. As shown in the literature, spectral analysis was employed to provide the evidence on the reduction of the external magnetic field according the strength of HBs [45]. Water’s properties of refractive index, viscosity and electric conductivity were significantly increased under applied magnetic fields [31,46]. More interestingly, as reported by Yoo et al. [47], electromagnetized AuNPs can mediate direct lineage reprogramming into induced dopamine neurons in vivo for Parkinson’s disease therapy. The experimental results demonstrated that the electromagnetic energy promotes the efficient conversion of somatic fibroblasts into induced dopaminergic neurons, both in vitro and in vivo.

Water plays an essential role in all living entities and functions as a solvent. In the human body, water plays the roles of a medium for nutrient transfer and a carrier for species, and stabilizing body temperature. Additionally, water participates in some biochemical reactions, such as hydrolysis [48,49], glycogen decomposition, and adenosine triphosphate (ATP) decomposition [50,51]. Water, a popularly used solvent, is generally considered an inert spectator in chemical reactions. However, liquid water with characteristic property of donor–bridge–acceptor for proton transfer and electron donating has been shown as a promising active reactant [52,53,54]. In addition, it is generally considered an independent reactant. As shown in the literature reporting the hydrogen evolution reaction (HER), the interaction energy of H_3_O^+^−OH^−^ is 46.9 kJ mol^−1^, but this required energy is increased ca. 2.5-times for H_3_O^+^ associating with additional four water molecules by HBs [55]. Moreover, gas water can catalyze many chemical reactions [56,57,58] via the formation of HBs with other molecules due to more free water molecules being available in its gas phase, compared to water in liquid phase with more-perfect tetrahedral symmetry of HB structure.

Surface plasmon resonance (SPR) excited the illuminated AuNPs to decay into energetic hot electrons. Hot electron transfer (HET) can promote many chemical reactions [59,60,61], including the dissociation of hydrogen [62], activation of oxygen [63], and electrocatalytic hydrogen evolution [64]. On the other hand, our group proposed an innovative method to create plasmon-activated water (PAW), which is prepared by treating deionized (DI) water with resonantly excited AuNPs [23,65,66]. Recently, the PAW-related papers, which are innovatively applicable in variously fields, are continuously published in our group [67,68,69,70,71,72,73,74,75,76,77,78,79]. Compared to DI water, the created additive-free PAW shows lower specific heat and boiling point [66], higher vapor pressure and osmosis [23], novel antioxidant and anti-inflammatory properties [23,67], and higher solubility and diffusion to solutes [23,65,67]. Based on these distinct properties, PAW can be served as source of green energy for improving the efficiencies of productions of hydrogen and oxygen via water splitting [65,69,72]. In green chemistry, PAW can be employed as a green reductant for production of AuNPs and AgNPs from their individual ions [66,67]. Also, it can extract more amounts of nutrients from tea leaves and *Polygonum multiflorum* (PM) [70]. The vapor from created PAW is an environmentally-friendly etchant that can modify surfaces of glass and wafers [71]. Polypyrrole electrode with a greater electroactive surface was electrochemically prepared by utilizing PAW [74]. In situ and real-time reduction of water molecules’ interaction for efficient water evaporation was performed in PAW solutions [75]. In addition, effectively reducing reagent concentrations can be achieved for electrochemical reactions in aqueous solutions using PAW [78]. SERS-active Au substrates with higher SERS enhancement and better signal reproducibility were prepared in PAW solutions [79]. PAW’s novel properties of enhancing solute diffusion and owning anti-inflammatory significantly shortens the time to remove uremic toxins during hemodialysis (HD) treatment, and makes HD safer [67,73]. In the meantime, it decreased fibronectin expression and attenuated renal fibrosis in a chronic kidney disease (CKD) mouse model [73]. An in vivo study of chronic sleep deprivation (CSD) rats, investigating the damage to liver structure and function from intense oxidative damage, was performed by feeding PAW instead of DI water [76]. Experimental results indicated that PAW markedly reduced oxidative stress with enhanced bioenergetics in hepatocytes. Moreover, it was demonstrated PAW dramatically induced a major antioxidative *Nrf2* gene in human gingival fibroblasts. This further confirms its cellular antioxidative and anti-inflammatory properties [77]. Furthermore, mice implanted with mouse Lewis lung carcinoma (LLC-1) cells drinking PAW alone, or together with cisplatin treatment, showed improved survival time compared to mice which drank DI water. Table 1 shows the different kinds of functional water reported in the literature. In addition, ongoing experiments indicate that PAW possesses high potential in therapeutic strategies for periodontal disease, Parkinson’s disease, and Alzheimer’s disease. Detailed descriptions on these innovative therapies on inflammatory-related CKD, CSD, and lung cancer based on PAW, are introduced in this review.

## 2. Results and Discussion

### 2.1. Creation and Characteristics of Plasmon-Activated Water (PAW)

As shown in our previous study [23], we reported an innovative and facile method for preparing small water-clusters (SWCs) with reduced-affinity HBs by letting bulk DI water flow through supported AuNPs under resonant illumination to give NP-treated (AuNT) water at constant temperature. Utilizing LSPR on illuminated AuNPs, the strong HBs of bulk water can be disordered when water is located at the illuminated Au/water interface. The prepared SWC is free of AuNPs. The energy efficiency for creating SWCs is ca. 17%. The resulting stable AuNT water exhibits distinct properties at room temperature, which are significantly different from the properties of untreated bulk water. Since the SWC (also the different term of AuNT water) was created from the HET, the term “SWC” (or term “AuNT water”) was replaced by the term “PAW” in the latest published papers.

**Figure 1:**
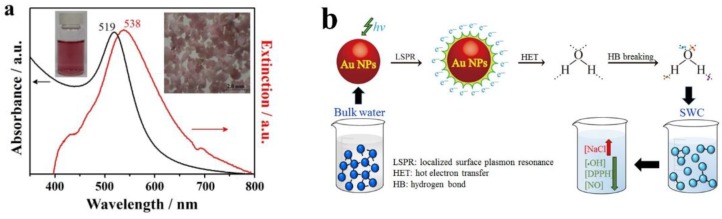
Preparation of plasmon-activated water (PAW) and its creation mechanism and main properties. (**a**) UV–vis absorption spectrum of AuNPs in solution (black line) and UV–vis extinction spectrum of AuNP-adsorbed ceramic particles (red line). (**b**) Schematics of mechanism of the preparation of SWC (also called PAW) with reduced hydrogen bonding based on LSPR effect on AuNPs under resonant illumination and distinct property and antioxidative activity of prepared SWCs. The formation of SWCs occurred at the resonantly illuminated Au interface. In storage and in the absence of AuNPs, both the forward reaction from bulk water to AuNT water and the corresponding reverse reaction from AuNT water to bulk water are slow, due to the huge activation energies required for both reactions. Therefore, the prepared SWC is temporarily trapped in a relatively stable state in an activation energy valley, in which it can persist in liquid water without reverting to normal hydrogen-bonding patterns observed in bulk water. The saturated solubility of NaCl in SWCs can be significantly increased. SWC itself can effectively scavenge free hydroxyl and DPPH radicals. Also, SWCs effectively reduces NO release from LPS-induced inflammatory cells [23].

As shown in Figure 1a, the SPR band of AuNPs in solution locates at ca. 519 nm. This spectral center is red-shifted to 538 nm, and becomes broader in the whole visible-light region for the AuNPs-adsorbed ceramic particles. This characteristic LSPR of AuNPs-adsorbed ceramics suggests that the effect of HET on breaking the HBs of bulk water is performed with illumination of white light. This phenomenon is further enhanced by wavelength-optimized resonant light, as used in this work with green light-emitting diodes (LEDs) at the wavelength maximum centered at ca. 530 nm. Figure 1b exhibits the schematics regarding the mechanism on the creation of SWCs with decreased hydrogen bonding due to the LSPR effect on AuNPs at resonant illumination, and the distinct properties and antioxidative activity of created SWCs. The creation of SWCs occurred at the resonantly illuminated AuNPs interface. After creation, both the forward reaction from bulk water to AuNT water and the corresponding reverse reaction from AuNT water to bulk water in absence of AuNPs are slow because the required activation energies for both reactions are very large. Thus, the created SWC is temporarily trapped in an activation energy valley with a relatively stable state in which SWC can maintain its energetic state, not reverting to the normal HB structure of bulk water. The measured saturated solubility of normal electrolyte of NaCl in SWCs is markedly enhanced. Also, the SWC itself is capable of effectively scavenging free hydroxyl and 2,2-diphenyl-1-picrylhydrazyl (DPPH) radicals, and effectively reduce NO release from lipopolysaccharide (LPS)-induced inflammatory cells [23].

**Figure 2:**
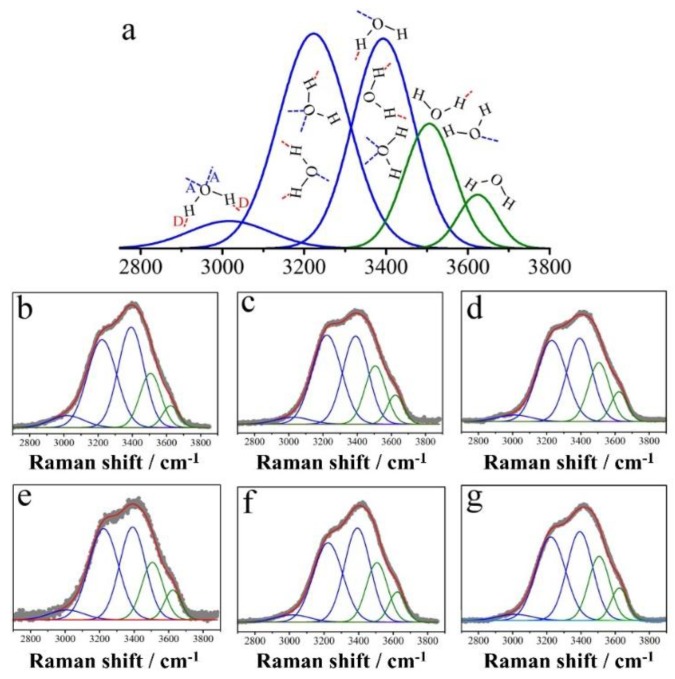
Assignments of five Gaussian components of OH stretching Raman bands and Raman spectra of OH stretching of various pure water and saline solutions. (**a**) In deconvolution, the five Gaussian components with the center wavenumbers at 3018, 3223, 3393, 3506, and 3624 cm^−1^ were adopted for all samples. The three components on the low frequency side are assigned to hydrogen-bonded water, while the remaining two high frequency side components are assigned to non-hydrogen-bonded water. The full width at half maximum (FWHM) of the individual component in the five-Gaussian fit was equal for all samples. These values are 234, 201, 176, 154, and 112 cm^−1^ for bands at 3018, 3223, 3393, 3506 and 3624 cm^−1^, respectively. Red capital letter D and blue capital letter A are noted as donor and acceptor of proton, respectively; (**b**) DI water for reference; (**c**) AuNT water (also called PAW) under illumination with fluorescent lamps in preparation; (**d**) sAuNT water (also called PAW) under illumination with green LED in preparation; (**e**) DI water with 0.9 wt % NaCl; (**f**) AuNT water with 0.9 wt % NaCl; (**g**) sAuNT water with 0.9 wt % NaCl [67].

Figure 2 demonstrates the OH-stretching Raman spectra and the corresponding assignments of five Gaussian components of OH stretching in Raman bands for various water samples. Further, the obtained Raman spectra were deconvoluted into five Gaussian sub-bands based on general methods demonstrated in the literature [80,81,82,83]. In this work, the band assignments are slightly different from those shown in the literature, the consistent rules are that the bands on the higher and the lower frequency sides are related to the weaker and the stronger hydrogen-bonded OH features, respectively. Therefore, the three components on the lower frequency side are assigned to hydrogen-bonded water; while the other two on the higher frequency side are assigned to non-hydrogen-bonded water. Further, the degree of non-hydrogen-bonded water (DNHBW) was defined as the ratio of the areas of the non-hydrogen-bonded OH stretching bands to the total areas of the stretching band, as shown in our previous report [67]. The calculated DNHBWs of DI, AuNT and super AuNT (sAuNT)-derived water are 21.29% (the value is 21.37% for DI water stored for 3 weeks), 25.07% and 26.78%, respectively. The very close values for as-prepared and stored DI water suggest 21.29% is a reliable blank value for bulk water employed in definition of DNHBW. Interestingly, this DNHBW was significantly increased, from 21.29% to 25.07% under the fluorescent lamp-irradiated LSPR effect on the supported AuNPs. This is a significant increase of 18% in DNHBW, which can be further enhanced to 26% under the green LED-irradiated LSPR effect. Moreover, the DNHBW values of DI, AuNT and sAuNT water with 0.9 wt % NaCl, which were termed as saline solution (DI), saline solution (AuNT) and saline solution (sAuNT), are 23.98%, 26.00% and 27.66%, respectively. Comparing pure water with its corresponding saline solutions, it was found that the DNHBW is markedly increased by 13% for DI water, but the increases are only 2.0% and 3.2% for saline solutions based on AuNT water and sAuNT water, respectively. However, the values of DNHBW of saline solutions (AuNT and sAuNT) are still significantly higher than that of DI water-based saline solution. As shown in the literature, almost tetrahedral structure of liquid water with two O–H bonds can be disrupted by the dissolving process of NaCl [84,85]. Different treatments on water confirm the LSPR effects with respect to the corresponding AuNT water owning different values of DNHBW [67].

**Figure 3:**
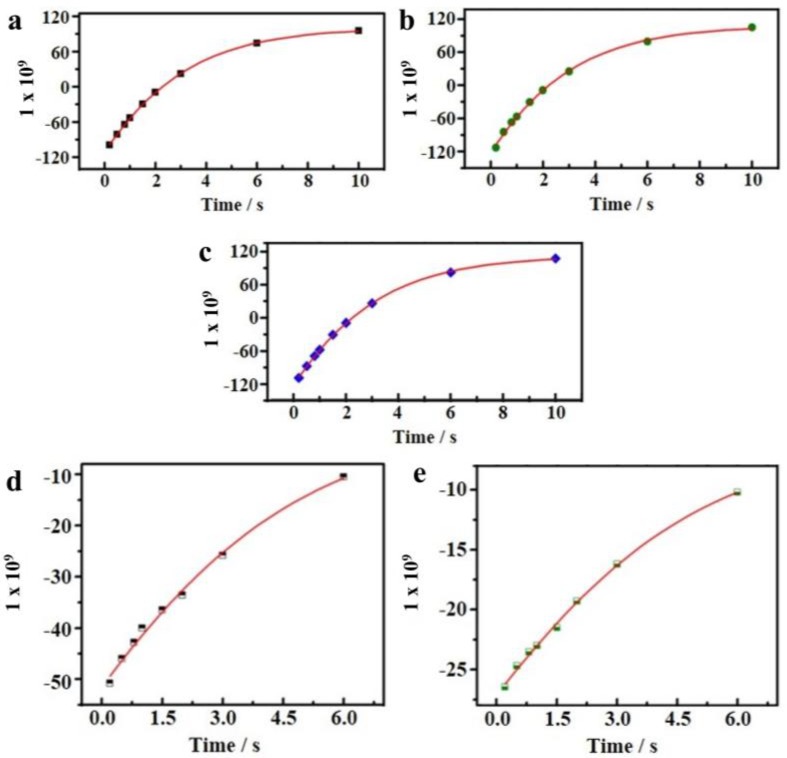
NMR T_1_ represents the time required for the longitudinal component of magnetization to recover its equilibrium value after applying a perturbing pulse sequence. Spectra (**a**–**e**) represent plus of spectral signals as a function of repetition time for DI water, AuNT (light-free) water, sAuNT water, D_2_O, and sAuNT D_2_O [66].

In addition, evidence for reduced HBs in sAuNT water was examined by using NMR relaxation time (T_1_) (Figure 3) [66]. Under magnetic field fluctuation, the relaxation time T_1_ of DI water is 3.092 s, shorter than 3.169 s of sAuNT water. Meanwhile, T_1_ of 3.087 s for AuNT water (light-free) is very closed to the value of DI water (Figure 3a–c). It demonstrates again that light illumination on supported AuNPs is necessary for the creation of treated water with reduced hydrogen-bonded structure. The relaxation time T_1_ of sAuNT water is 1.029 ± 0.0035 times longer than that of DI water (*n* = 3). The longer relaxation time indicates the lower degree of hydrogen-bonded interaction between water molecules in sAuNT water, resulting in reducing the effective proton transfer between spins and lattice. The T_1_ of 3.018 s (*n* = 2) for DI water solution containing 50 wt % of sAuNT water is between values of DI water and sAuNT water. It suggests that the DNHBS in water is tunable. In addition, the longer relaxation time of ca. 5.580 s is also observed on the sAuNT-treated D_2_O (Figure 3d,e), compared to untreated D_2_O (ca. 5.063 s). These analyses on Raman spectra and NMR relaxation time suggest the intrinsic reduction of hydrogen-bonded structure in sAuNT water after our proposed innovative process [66]. Also, as reported in the literature [86,87], electrons would correspondingly respond in the magnetic field. Therefore, we found that the duration of metastable electron-doping sAuNT water can be effectively prolonged in external magnetic fields [66].

### 2.2. Physchemical and Cellular Antioxidation Properties of PAW

**Figure 4:**
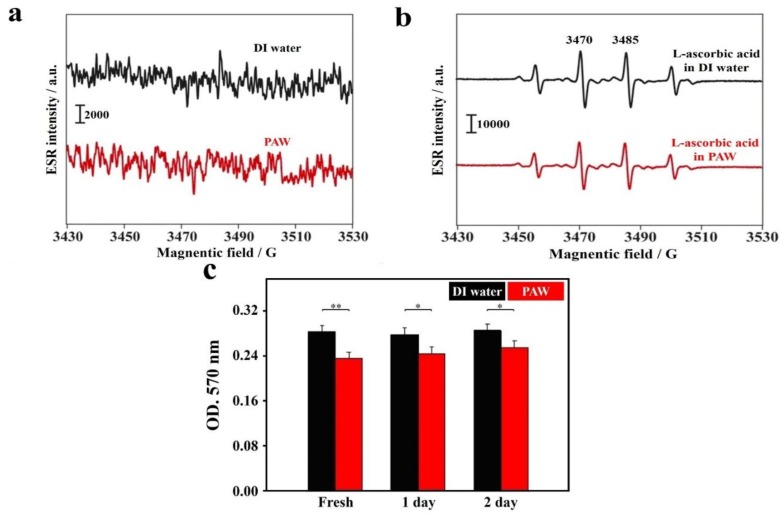
ESR spectra of hydroxyl free radicals and antioxidative effects based on DI water and PAW. (**a**) Spectra of DI water (black line) and PAW (red line) for reference; (**b**) Spectra of DI water plus the antioxidant l-ascorbic acid (black line) and PAW plus l-ascorbic acid (1.775 µM) (red line). Hydroxyl free radicals were obtained using the well-known Fenton reaction, in which ferrous iron donates an electron to hydrogen peroxide to produce a hydroxyl free radical; (**c**) Antioxidative effect of PAW to H_2_O_2_. The OD at 570 nm of H_2_O_2_ (2.5 nm) prepared in DI and PAW waters. The corresponding *p* values are 0.00491, 0.0233, and 0.0357 for PAW after its preparation for 0, 1, and 2 days, respectively [77].

As reported in the literature, hydroxyl radicals are the most cytotoxic ROS, and as such, they can directly or indirectly damage DNA and cause cancer [88,89,90]. It is well known that excessive amounts of ROS are produced at sites of inflammation. Therefore, the unique ability to scavenge free hydroxyl radicals and other distinct properties of PAW compared to DI water may offer a new therapy on suppressing inflammation and even on curing cancer. Figure 4a demonstrates the electron spin resonance (ESR) spectra regarding hydroxyl radicals of DI water and PAW for reference. No significant peaks were observed for either DI water or PAW. This result suggests that the created electron-doping PAW differs from the reported engineered water nanostructures with a very strong surface charge, which demonstrated strong signals of hydroxyl radicals in an ESR spectrum [91]. Figure 4b demonstrates the ESR spectra regarding hydroxyl radicals of DI water, plus the known antioxidant, l-ascorbic acid [43], and PAW plus l-ascorbic acid, in the well-known Fenton reaction, as described in the experimental section. The four ESR splitting signals shown in these spectra are characteristic of hydroxyl radicals [13,43]. Interestingly, the production of hydroxyl radicals was significantly reduced in the PAW-based system compared to the DI water-based system with l-ascorbic acid. The corresponding ESR average intensities of the two strongest peaks at ca. 3473 and 3488 G in the PAW-based system significantly decreased by ca. 21% (** *p* < 0.01), compared to that for an experiment performed in the DI water-based system. Furthermore, in the Fenton reaction, free hydroxyl radicals are generated from hydrogen peroxide (H_2_O_2_). H_2_O_2_ is one of the products of reactions catalyzed by oxidase enzymes in many biological and environmental processes. However, H_2_O_2_ is also one kind of ROS that can cause functional and morphological disturbances, as well as cancer, when produced in excess in the human body. It was demonstrated that H_2_O_2_ is a reservoir for generating HOx by reacting with OH radicals (Equation (1)) [92,93]. Water was shown to be favorable for its catalytic effect on radical–radical (H_2_O_2_–OH) reactions due to the ability of water to form stable complexes (HO_2_·H_2_O) with HO_2_ radicals through hydrogen bonding.

H_2_O_2_ + OH → HO_2_ + H_2_O (in the atmosphere) (1)

HO_2_ + H_2_O ↔ HO_2_·H_2_O (in the atmosphere) (2)

In the presence of liquid water, the oxidation of H_2_O_2_ becomes more complex by the following three steps [57].

H_2_O_2_·H_2_O + OH → HO_2_ + 2H_2_O (3)

H_2_O_2_ + H_2_O·OH → HO_2_ + 2H_2_O (4)

H_2_O_2_·OH + H_2_O → HO_2_ + 2H_2_O (5)

Either in the atmosphere or in an aqueous solution, water deeply dominates the equilibrium of these reactions. In a previous study, it was reported that PAW provides more available sites for forming HBs [23]. In addition, compared to bulk water, which is recognized as being constructed of numerous large-sized water clusters, PAW with reduced HBs forms smaller water clusters, and thus, presumably has more active sites. According to Le Chatelier’s principle, the forward reactions of Equations (2)–(5) dramatically occur, accompanied by consumption of quantities of H_2_O_2_ and OH free radicals when DI water is replaced by PAW. Therefore, PAW might consume H_2_O_2_ during the Fenton reaction. The evidence of scavenging H_2_O_2_ by PAW was examined using an H_2_O_2_ assay kit (Figure 4c). The optical density (OD) at 570 nm for H_2_O_2_ (2.5 nmol), prepared using DI water, was 0.284 ± 0.010. This value decreased to 0.235 ± 0.011 as DI water was replaced by PAW, meaning nearly 17.2% of the H_2_O_2_ had been consumed by PAW. Also, the above ESR result demonstrated that PAW plus l-ascorbic acid can reduce more than 21.0% of the hydroxyl radicals from the Fenton reaction than DI water plus l-ascorbic acid can. The source of hydroxyl radicals was from H_2_O_2_, and 17.2% of H_2_O_2_ was consumed by PAW. In addition to the effect of PAW on H_2_O_2_, PAW plus l-ascorbic acid reduced more than 4.2% of the hydroxyl radicals than DI water plus l-ascorbic acid did. This means that a synergetic effect occurred between PAW and l-ascorbic acid. To the best of our knowledge, this enhanced antioxidant activity of scavenging free radicals in a PAW-based system, instead of a conventional DI water-based system, is the first report in the literature [77]. Additionally, the ability of PAW to scavenge H_2_O_2_ was slightly reduced with time.

**Figure 5:**
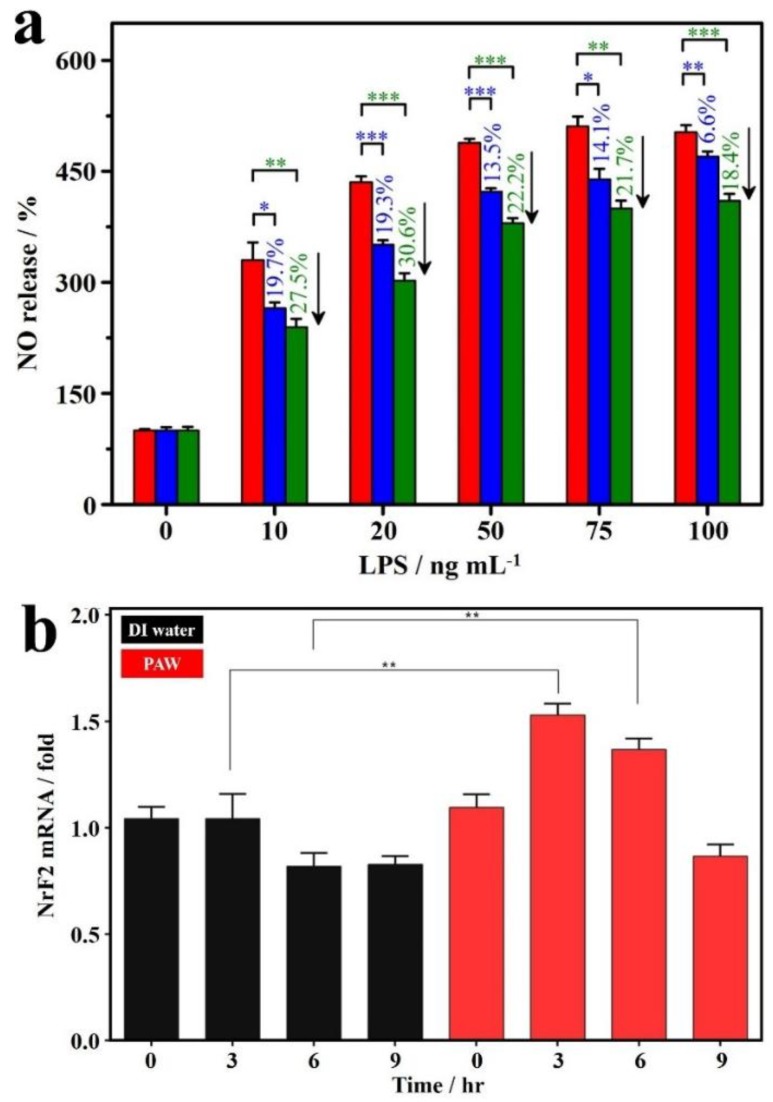
Antioxidative activities of PAW based on cellular experiments. (**a**) Antioxidative activity of AuNT water (also called PAW, blue block) and sAuNT water (also called PAW, green block) compared to DI water (red block) on reduction of lipopolysaccharide (LPS)-induced NO release with dose of LPS. DI water, AuNT water and sAuNT water were used for medium preparation. * *p* < 0.05; ** *p* < 0.01; *** *p* < 0.001. (**b**) Induction of *Nrf2* expression in human gingival fibroblasts (HGFs) exposed to PAW. HGFs were incubated in culture medium prepared with DI water or PAW for 0, 3, 6, and 9 h. *Nrf2* mRNA expression levels were quantified by a real-time PCR, and results are presented as the relative normalized expression with *GAPDH*. Data were analyzed by Student’s *t*-test, and results are presented as the mean ± SD. ** *p* < 0.01. The corresponding *p* values are 0.00521 and 0.00453 for 3 and 6 h, respectively [67,77].

As shown in the literature [94,95], evaluation on the inhibiting abilities using an lipopolysaccharide (LPS)-activated monocyte/macrophage nitric oxide (NO) system was generally performed for biological studies focusing on anti-inflammatory effects. Figure 5a displays the inflammation-preventive effects of created AuNT water and sAuNT water, as compared to DI water, regarding the reduction of LPS-induced NO release. Clearly, the increased levels of NO productions were significantly reduced (*p* < 0.05) in AuNT water (especially for sAuNT water prepared under resonant LED illumination)-DMEM (Dulbecco’s Modified Essential Medium) in the presence of LPS from 10 to 100 ng mL^−1^. Incubation in created AuNT water-DMEM suppresses the NO release in LPS-activated macrophage cells [67]. Since *Nrf2* is a well-known anti-oxidative gene that can prevent damage to cells from ROS, the role of PAW on the expression of the *Nrf2* gene was further examined to investigate its distinct anti-oxidative property. In experiments, human gingival fibroblasts (HGFs) were exposed to cultured media based on DI water or PAW for 0, 3, 6 and 9 h. Then messenger (m)RNA expression levels of *Nrf2* were measured by a real-time polymerase chain reaction (PCR). As displayed in Figure 5b, the levels of the mRNA expressions of *Nrf2* in HGFs were markedly induced by PAW with exposure for 3 and 6 h. Then the level was decreased to a normal level after exposure for 9 h. This result indicates the potential role of PAW on the defense to the oxidative stress through induction of *Nrf2* gene [77].

A previous study showed that *Nrf2* is a transcription factor that responds to oxidative stress by binding to the ARE in the promoter of antioxidant enzyme genes, such as NAD(P)H: quinone oxidoreductase 1, glutathione *S*-transferases, and glutamate cysteine ligase [96]. Activation of the *Nrf2* pathway by sulforaphane, a phytochemical, was well documented and linked to cancer chemoprevention [13]. Similarly, curcumin, a well-known polyphenol, was also reported to induce *Nrf2*, and had an antioxidant response [97]. PAW may have similar properties to these antioxidant substances. Therefore, the exact molecular mechanism based on PAW requires further investigation. Although inflammation is one of the major defense mechanisms against infection and in the repair of injured tissues, prolonged chronic inflammation may also contribute to the development of various chronic and neoplastic diseases in humans. The development of nanotechnology and nanomaterials with anti-inflammatory properties is rapidly being exploited, and the anti-inflammatory potential of PAW is, therefore, worth further evaluating. Therefore, we demonstrated that PAW increased *Nrf2* expression, one of the defense mechanisms against the ROS-induced cellular stress response in HGFs. Additionally, administration of PAW can be developed into an alternative strategy for treating chronic diseases, such as non-small cell lung cancer (NSCLC), which is related to local chronic inflammation [77].

### 2.3. Innovative Therapeutic Strategy Utilizing PAW

**Figure 6:**
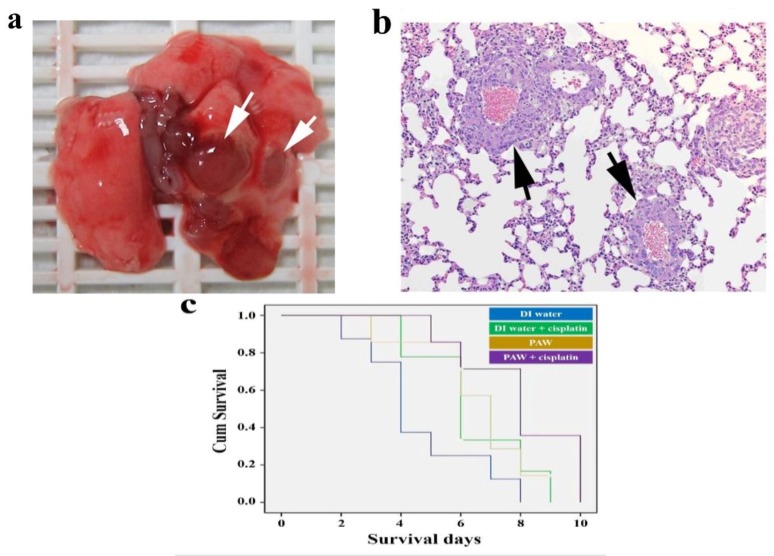
Pathological features and survival curve on the LLC-1-xenograft mice. (**a**) Lung metastasis in LLC-1-xenograft mice: gross observation of the whole lung (arrows); (**b**) Lung metastasis in LLC-1-implanted mice, HE staining (right, 200× magnification) of metastatic tumor lesions (arrows); (**c**) The overall survival time (days) of LLC-1-implanted mice treated with DI water (*n* = 9), DI water plus cisplatin (*n* = 8), PAW (*n* = 7), or PAW plus cisplatin (*n* = 7) [77].

Gross observations of whole lungs to lung metastasis in LLC-1 xenograft mice are shown in Figure 6a. All tumor-like lesions were identified on lung lobes and thoracic walls, but not present in other organs of thoracic and abdominal cavities. These tumor-like lesions were further identified by hematoxylin and eosin staining as LLC-1 tumor lesions (Figure 6b). As shown in Figure 6b, the LLC-1 tumor lesions localized around blood vessels suggested that the injected LLC-1 cells invaded pulmonary tissues via circulation. The metastasis rate of LLC-1 cells was calculated according to gross observations of the LLC-1 lung tumor presence, and was analyzed by a two-tailed Fisher’s test. Interestingly, five of 17 LLC-1 grafted mice drinking DI water demonstrated lung metastasis, compared to zero of the 14 LLC-1 grafted mice drinking PAW. The metastasis rate in PAW-consuming mice was significantly lower than that of DI water-consuming mice. The average survival time of PAW-fed mice was 6.57 ± 0.66 days, whereas in DI water-fed mice, it was 4.62 ± 0.71 days. In cisplatin-administrated mice, PAW-fed mice also had a prolonged survival time of 8.01 ± 0.77 days, compared to 6.38 ± 0.61 days for DI water-fed mice (Figure 6c). This result suggests that PAW may enhance the tumor suppression efficiency of cisplatin in LLC-1-implanted mice. This can be attributed to the different states of cisplatin in DI water and in PAW. It was reported that cisplatin is poorly soluble in water [98], indicating some aggregations of cisplatin molecules are generated in DI water [77].

**Figure 7:**
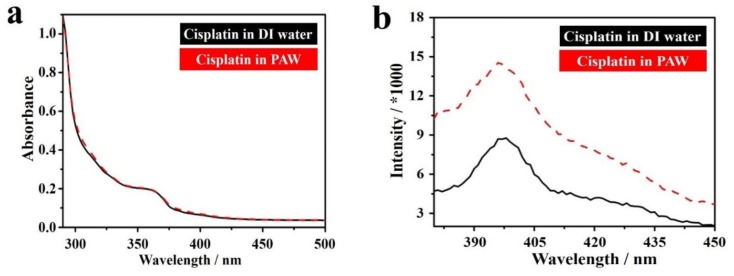

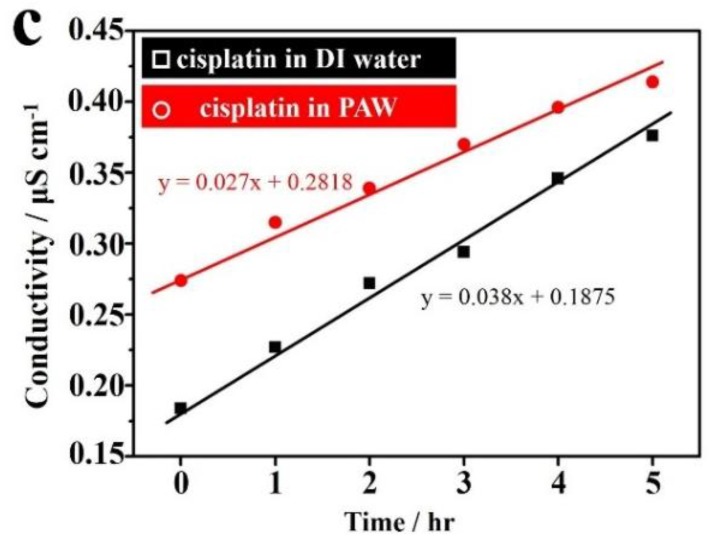
Conformation of cisplatin in DI water and PAW. (**a**) The absorption spectra of cisplatin in DI water and PAW. (**b**) The PL spectra of cisplatin in DI water and PAW with an excitation wavelength of 350 nm. (**c**) The conductivities of cisplatin solutions in DI water and PAW with time [77].

The absorption spectra showed the OD at 362 nm of cisplatin in PAW was almost the same as that in DI water (Figure 7a). However, a significant difference was observed in photoluminescence (PL) spectra with an excitation wavelength of 350 nm (Figure 7b). Cisplatin displayed emission bands at 396 and 397 nm in DI water and in PAW, respectively. The PL intensity of cisplatin in PAW was 1.6-fold higher than that in DI water. This evident difference can perhaps be attributed to the status of cisplatin complexes in the different waters. The poor solubility of cisplatin in DI water results in the formation of some aggregations that quenched the fluorescence. However, this phenomenon was observed less, because cisplatin can be more easily dissolved in PAW. The solubilities of cisplatin in DI water and in PAW were measured at 25 °C. The solubility of cisplatin in PAW was 3.4 ± 0.11 mg mL^−1^, which was higher than that in DI water (2.6 ± 0.01 mg mL^−1^). The increased solubility was ca. 30.8%, indicating PAW improved the solubility of cisplatin. This reveals that PAW improved the solubility of cisplatin and reduced interactions among cisplatin molecules, thus showing a higher PL intensity. Compared to the aggregated cisplatin in DI water, which could be considered to be a large size and of high molecule weight, well-dispersed cisplatin in PAW could be transported more easily across plasma membranes, thus enhancing the tumor suppressive efficiency of cisplatin in LLC-1-implanted mice. Furthermore, the zeta potentials of cisplatin solutions with 0.5% sodium chloride (NaCl) were also monitored over time. Charges of the cisplatin solutions were −8.6 and −19.3 mV with DI water and with PAW, respectively. Moreover, the negatively charged environment was stable for the following 2 days. A negatively charged environment is favorable for maintaining the activity of cisplatin before it is transported across plasma membranes [99]. The activity of cisplatin was mainly dominated by the stability of cisplatin. It had been reported that cisplatin was easily hydrolyzed [100]. The hydrolysis process released two chloride ions into water. The presence of chloride ions in water would increase the solution conductivity. To evaluate the stability of cisplatin in DI water and in PAW, the cisplatin solutions (0.28 mM) were prepared, and the conductivities were measured with time at 25 °C (Figure 7c). The conductivity of fresh cisplatin solution in PAW (0.274 μS cm^−1^) was higher than that in DI water (0.184 μS cm^−1^). Mindfully, the higher conductivity of cisplatin solution in as-prepared PAW was not attributed to the higher degree of cisplatin’s hydrolysis, due to the intrinsically high conductivity of PAW. In storage with time, the conductivities of both solutions increased gradually, indicating that the cisplatin were hydrolyzed in both solutions. By plotting the relation of conductivity to time, two linear plots were obtained from DI water-based cisplatin and PAW-based cisplatin solutions. The slope of PAW-based cisplatin solution was 0.027, which was lower than that of DI water-based cisplatin solution (0.038). This indicated that the use of PAW could avoid hydrolysis of cisplatin, thus enhancing its stability. The high stability of cisplatin in PAW could express the high activity of cisplatin in LLC-1 further. Therefore, higher cisplatin activity could be maintained when it was dissolved in PAW [77].

**Figure 8:**
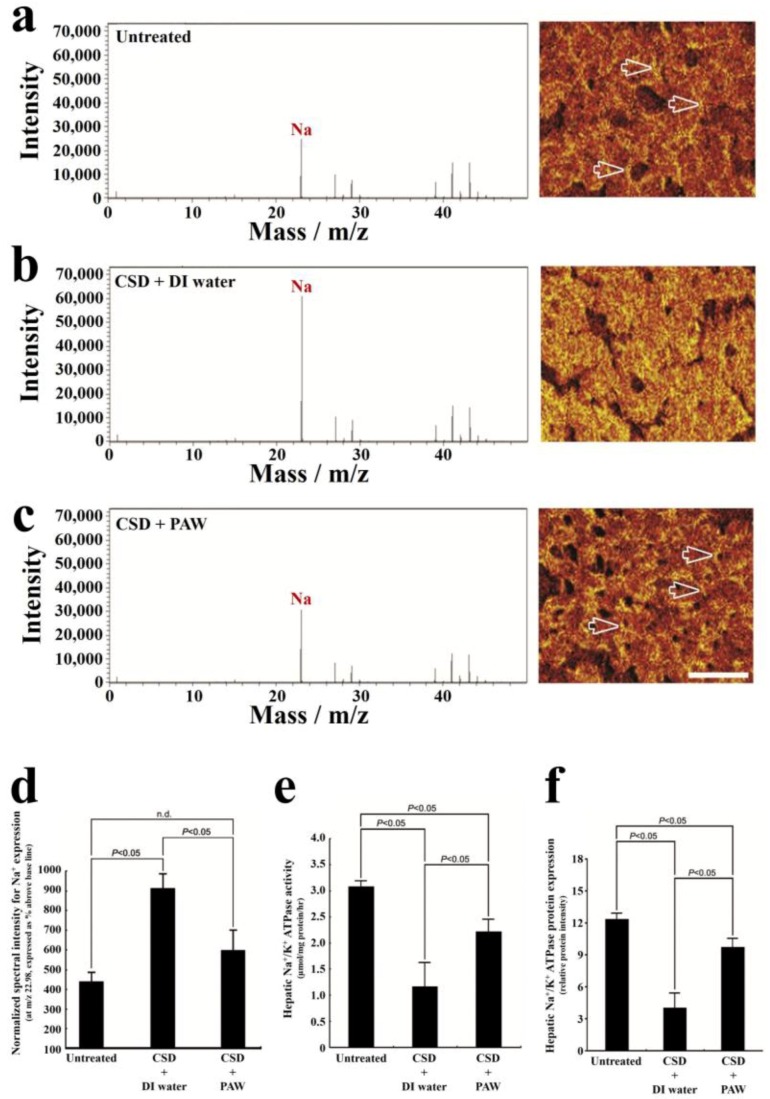
Effects of PAW on preserving Na^+^/K^+^ ATPase function and restoring transmembrane ionic gradient following CSD injury. Positive spectra/ionic images showed that in normal untreated rats, most of the Na^+^ signals were localized to the extracellular portion of the hepatocyte (arrows in (**a**)). Following CSD, strong Na^+^ signals were detected in the cytoplasmic portion of the hepatocyte (**b**), indicating the impairment of transmembrane ionic regulation (**b**). However, in animals that drank PAW daily during the entire CSD period, the distribution pattern of Na^+^ was very similar to that of normal untreated ones, in which the majority of Na^+^ were localized to the extracellular sinusoid space (arrows in (**c**)). The corresponding data of the normalized spectral intensities for (**a**–**c**) (**d**). Biochemical data coincided well with ionic imaging findings in which PAW effectively preserved hepatic Na^+^/K^+^ ATPase expression (**f**) and improved Na^+^/K^+^ ATPase activity (**e**). Scale bar = 100 μm in (**a**–**c**). n.d. represents non-delivery [76].

As reported in the time-of-flight secondary ion mass spectrometry (TOF-SIMS), PAW can effectively relieve hepatic oxidative damage that resulted from chronic sleep deprivation (CSD) [76]. In normal untreated rats, most of the Na^+^ signals were distributed in the extracellular portion (i.e., hepatic sinusoids) of hepatocytes, as shown with arrows in Figure 8a. In contrast, strong Na^+^ signals were accumulated in the intracellular portion of hepatocytes, following CSD, as shown in Figure 8b. The enhancing cytoplasmic Na^+^ expression suggests an intracellular Na^+^ overload, which could lead to ionic dyshomeostasis. Consequently, this contributes to the progresses of cytosolic acidification and metabolic deficiencies [101]. However, for rats subjected to CSD and daily drinking PAW instead of normal DI water, the hepatic Na^+^ expression successfully returned to nearly normal levels, in which the majority of Na^+^ ions was distributed in the extracellular portion of hepatocytes , as shown with arrows in Figure 8c. These ionic images (Figure 8a–c) and the normalized spectral intensity (Figure 8d) are well consistent with the results of the hepatic Na^+^/K^+^ ATPase activity and protein assay, as shown in Figure 8e,f. The impairment (or recovery) of ionic homeostasis paralleled the reduction (or increment) in Na^+^/K^+^ ATPase activity. These consistent findings suggested that daily drinking PAW during CSD is capable of effective preservation of Na^+^/K^+^ ATPase function and can successfully recover the corresponding ionic gradient to a normal stage.

**Figure 9:**
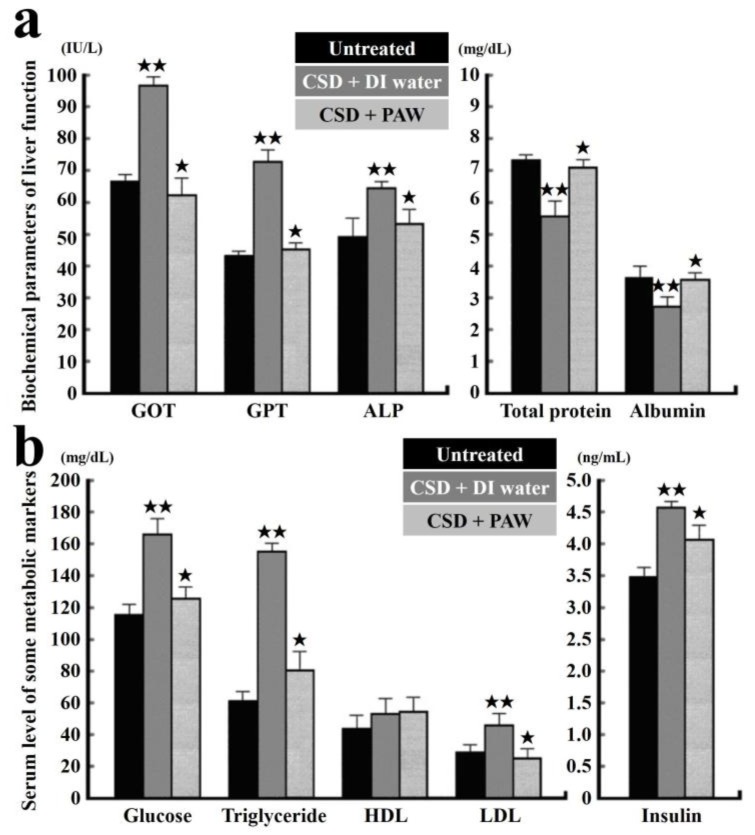
Histograms showing the serum level of biochemical markers related to liver (**a**) and metabolic (**b**) functions. Note that CSD contributes to severe liver and metabolic deficiencies. However, drinking of PAW successfully exerts beneficial effects on liver and metabolic function, in which almost all biochemical markers were noticeably returned to nearly normal values. ^★^ *p* < 0.05; ^★★^ *p* < 0.01 [76].

In addition, GOT, GPT, ALP and albumin/total protein were employed to evaluate the liver function of normal untreated and CSD rats drinking PAW or DI water. The results revealed that in normal untreated rats, the measured concentrations of GOT, GPT and ALP were all within normal ranges, as expected (Figure 9a). Following CSD and daily drinking DI water, impairment of liver and metabolic functions was obviously shown by increased levels of GOT, GPT and ALP. Also, the incidences of hyperglycemia, hypertriglyceridemia, hyperlipidemia and hyperinsulinemia are markedly increased (Figure 9b). Nevertheless, in rats subjected to CSD and daily drinking PAW, both the metabolic and the liver functions gradually restored to nearly normal values (Figure 9a,b) [76].

**Figure 10:**
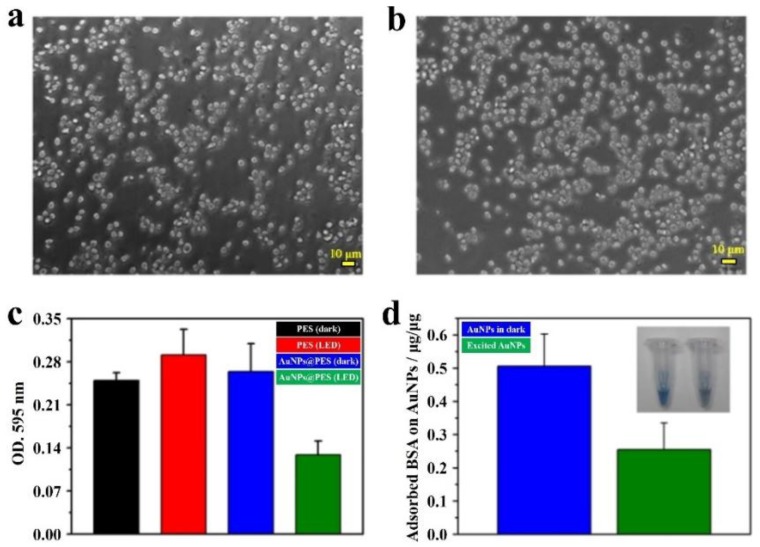
The cytocompatibility and hemocompatibility of excited AuNPs@PES membranes. Microscopic images of the RAW 264.7 macrophage cell line growing on (**a**) a blank plate in the dark and (**b**) an excited AuNPs@PES membrane for 4 h. (**c**) Tests of protein adsorption based on BSA. (**d**) Adsorption of BSA onto AuNPs under resonant illumination and in the dark (*n* = 3). Inset: Images of AuNP-containing solutions under resonant illumination (**right**) and in the dark (**left**). The precipitated AuNPs were dispersed in Bio-Rad Protein Assay solutions after shaking a mixed solution of AuNPs and BSA for 1 h [73].

In study on the multifunctions of excited AuNP-decorated artificial kidney (AK) with efficient hemodialysis (HD) and therapeutic potential [73], except the removal efficiency to uremic toxin, the test of biocompatibility was further studied to evaluate the possible application in further clinical HD. A PES membrane, which is the major component of AKs, was employed as the template for loading of AuNPs. In this work, AuNPs were coated on one side of the membrane (AuNPs@PES); while the other side of the membrane is free of AuNPs. The viability of RAW 264.7 cells on the AuNPs@PES was further examined using morphological criteria based on an optical microscope (Figure 10a,b). The cell culture experiments were performed under resonant illumination for 4 h. In addition, the AuNPs-coated membrane in one side did not contact the cells directly. The majority of RAW 264.7 cells on the AuNPs@PES (LED) membrane demonstrated a typical circular shape, which was similar to that shown in the control group without further membrane treatment. This result indicated that the experimental cells were alive in a healthy state. Moreover, as the cell culture time extended to 6 h, the cell viability in the AuNPs@PES (LED) was not obviously changed, as compared to that observed in the control group. It indicated that the AuNPs@PES membranes were biocompatible. Similar study on the biocompatible applications of AuNPs in medicine was also reported in the literature [102]. Thus, the AuNP-coated membrane reported in this work is noncytotoxic. In addition, RAW 264.7 cells did not directly contact the AuNPs. This also promised the noncytotoxic PAW was prepared between the interface of the AuNPs-coated membrane and water under resonant illumination during cell culture. In HD, protein adsorption occurred as the HD membrane first contacting the blood, which is known as the main factor that can cause deadly thromboses. Thus protein adsorption was examined employing bovine serum albumin (BSA) to investigate the hemocompatibility of the prepared AuNPs@PES membranes. Figure 10c shows the concentrations of absorbed BSA, which were measured using an optical instrument of enzyme-linked immunosorbent assay (ELISA) reader at 595 nm. The quantity of adsorbed BSA on PES membranes in dark was close to that for the experiment performed under illumination. This revealed that the green LED did not influence the protein adsorption. Meanwhile, the obtained value from the experiment based on AuNPs@PES membranes (in dark) also revealed that the capacity in adsorption of BSA was independent of the coating of AuNP because BSA did not directly contact the AuNPs. Encouragingly, the adsorption of BSA was significantly decreased when the AuNPs@PES membrane was resonantly illuminated with green LEDs. In addition, the measured optical density dropped ca. 51.1% compared to the AuNPs@PES membrane (in dark). This phenomenon is ascribed by two factors. First, it is recognized that proteins adsorbed on membranes are resulted from hydrophobic interactions, hydrogen bonding and electrostatic attraction [103]. Also, PAW with reduced HBs can provide more available sites to form HBs with other species. PAW can form HBs with proteins and membranes, thus, it respectively reduce the chances in formation of HBs within proteins and membranes. Moreover, hot electrons were released at resonantly-illuminated AuNPs. This produced a negatively charged environment in the vicinity of AuNPs@PES membranes. Thus, this negatively charged AuNPs@PES membrane demonstrated electrostatic repulsion to negatively charged proteins. It decreased the adsorption of BSA. The direct evidence was further examined in solutions (1 mL) containing AuNPs (0.5 mL, 873 ppm) and BSA (0.5 mL, 2 mg/mL) under resonant illumination and in dark for reference. The adsorbed BSA on AuNPs was quantified by assaying the free BSA in the solution based on its standard calibration curve. The free BSA was obtained by centrifugation at 13,000 rpm. After shaking at 100 rpm in dark for 1 h, it was measured and the calculated value for BSA adsorbed by 1 μg of AuNPs was 0.51 ± 0.09 μg (Figure 10d). Interestingly, the adsorbed BSA on 1 μg of AuNPs was significantly reduced to 0.26 ± 0.08 μg (a half lower) as the experiment was performed under resonant illumination. In experiments, the precipitated AuNPs after centrifugation were taken out and redispersed in a Bio-Rad Protein Assay solution. In addition, the color of the AuNP-containing solution for the experiment performed in dark was bluer than that for the experiment performed under resonant illumination (inset in Figure 10d). This means that more BSA was adsorbed on AuNPs for the experiment performed in dark. These experimental results indicate that excited AuNPs can effectively avoid the adsorption of BSA because of the electrostatic repulsion between AuNPs and BSA both with negative charges.

**Figure 11:**
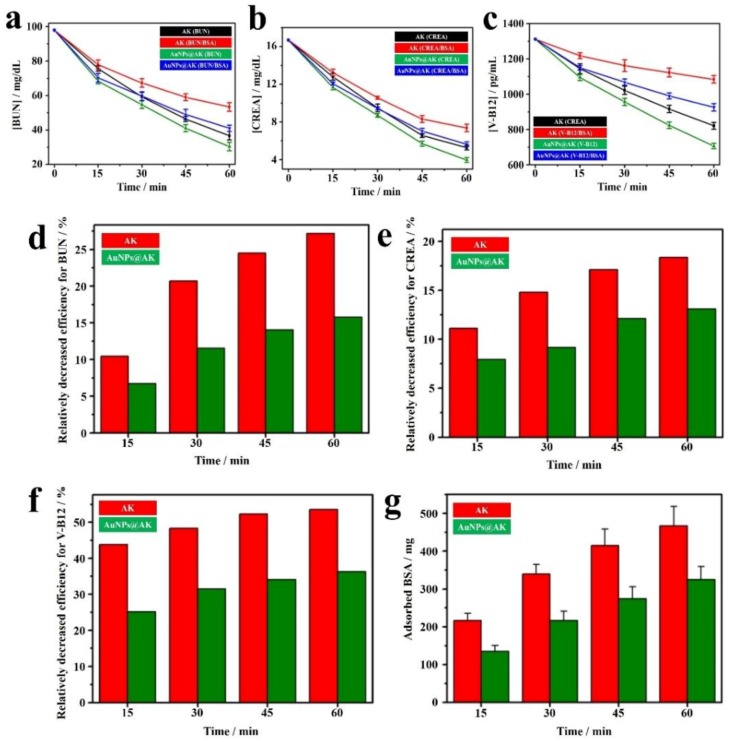
Influence of protein adsorption on the efficiencies of removing uremic toxins for an artificial kidney (AK) and excited AuNPs@AK. Flow rates for injections of the sample and saline solutions were 14 and 20 mL min^−1^, respectively. Removal efficiencies of (**a**) BUN; (**b**) CREA; and (**c**) V-B12 in the absence and presence of BSA (4 mg mL^−1^) (*n* = 3); The relative removal efficiencies of (**d**) BUN; (**e**) CREA; and (**f**) V-B12 in the absence and presence of BSA (4 mg mL^−1^); (**g**) The amount of adsorbed BSA onto the AK and excited AuNPs@AK during hemodialysis [73].

Figure 11a–c demonstrate the efficiencies on removing blood urine nitrogen (BUN), creatinine (Crea) and V-B12 in experiments with and without BSA [73]. Average values of 22.8%, 39.5%, 52.7% and 62.6% of BUN can be removed at 15, 30, 45 and 60 min, respectively, for the illuminated AK system without BSA. However, corresponding average values of BUN removals were reduced to 20.4%, 31.3%, 39.8% and 45.6% at the same sampling times in the presence of BSA (4 mg/mL). The relatively decreased efficiencies for BUN removals were 10.4%, 20.7%, 24.5% and 27.1%, respectively, as shown in Figure 11d. In calculation, the relatively decreased efficiency for BUN removal was defined as the following equation:

relatively decreased efficiency/% = [(*D* − *D*_BSA_)/*D*] × 100 (6)

For examine, the average BUN concentration decreased 22.8 mg/mL without BSA (for D) and this value decreased 20.4 mg/mL with BSA (for DBSA) at 15 min. According to Equation (6) the relatively decreased efficiency was 10.4%. At the same time, the adsorbed BSA on the AK were ca. 216.3, 339.9, 415.1 and 467.5 mg at sampling times of 15, 30, 45 and 60 min, respectively (Figure 11g). The obtained results confirm our hypothesis that the adsorbed BSA would create difficulties for the removal of uremic toxins of BUN, CREA and V-B12 (Figure 11a−f). The decrease of the efficiency in removing uremic toxins with an increase of the amount of adsorbed BSA was also observed for experiment based on the excited AuNPs@AK (Figure 11d−f). Nevertheless, compared to the system using AuNPs-free AK, the efficiencies in removing BUN based on the excited AuNPs@AK in the absence of BSA were increased by 36.4%, 12.8%, 11.5% and 11.4% at sampling times of 15, 30, 45 and 60 min, respectively. Interestingly, these increases were further magnified to corresponding 47.4%, 22.6%, 22.5% and 24.4% in the presence of BSA (Figure 11d). These results can be ascribed to the quantities of BSA adsorbed onto the excited AuNPs@AKs were relatively lower, as shown in Figure 11g. Figure 11e,f demonstrate the consistent patterns in removing CREA and V-B12, respectively. Conclusively, HD efficiencies based on excited AuNPs@AKs were further markedly improved, as compared to the conventional AK system, in the presence of BSA, which was similar to the real HD conditions. Moreover, in the study on evaluation of illuminated AuNPs@AK in CKD mice in vivo, quantification of fibronectin-labeled glomeruli in the kidneys suggested that daily drinking PAW markedly decreased fibronectin aggregation compared to that in DI water-drinking mice [73]. In addition, the thickness of Bowman’s capsules from CKD mice drinking PAW, was thinner than that of CKD mice drinking DI water. These interesting findings indicated that PAW created in the inner channel of excited AuNPs@AKs indeed prevents CKD mice from suffering renal fibrosis. Further clinical trial utilizing PAW is expecting in the future.

## 3. Conclusions and Future Perspectives

We report an innovative and facile method for preparing PAW with intrinsically reduced-affinity HBs, by letting bulk water flow through supported AuNPs under resonant illumination to give NP-treated (AuNT) water at constant temperature. The energy efficiency for creating PAW is ca. 17%. The resulting stable and energetic mass-production PAW exhibits distinct properties at room temperature, which are significantly different from the properties of untreated bulk water, examples being their ability to scavenge free hydroxyl and 2,2-diphenyl-1-picrylhydrazyl radicals and to effectively reduce NO release from lipopolysaccharide-induced inflammatory cells. The analyses of Raman spectra and NMR relaxation times suggested the intrinsic reduction of HB structures in PAW. In addition to the effect of PAW on scavenging H_2_O_2_, PAW plus l-ascorbic acid reduced more than 4.2% of the hydroxyl radicals than DI water plus l-ascorbic acid did. This means that a synergetic effect occurred between PAW and l-ascorbic acid. In addition, inflammation-preventive effects of PAW were observed, compared to DI water, with respect to the reduction of LPS-induced NO release. Also, experimental result suggests a potential role of PAW on the oxidative stress defense through *Nrf2* gene induction.

In this review, we showed PAW can decrease fibronectin expression and attenuate renal fibrosis in a CKD mouse model. Experimental results of in vivo experimentation on CSD rats indicated that PAW markedly reduced oxidative stress with enhanced bioenergetics in hepatocytes. Moreover, it was demonstrated PAW dramatically induced a major antioxidative *Nrf2* gene in human gingival fibroblasts, which further confirms its cellular antioxidative and anti-inflammatory properties. Furthermore, mice implanted with mouse Lewis lung carcinoma (LLC-1) cells drinking PAW alone, or together with cisplatin treatment, showed improved survival time and less metastasis compared to mice which consumed only DI water.

Furthermore, the ongoing animal disease models disclosed that the Alzheimer’s mice treated with PAW present the better memory performance, and little amyloid and phosphorylated tau burden in hippocampus. PAW can effectively preserve tyrosine hydroxylase expression in the substantia nigra of 1-methyl-4-phenyl-1,2,3,6-tetrahydropyridine (MPTP)-induced Parkinsonism rats. Periodontally diseased rats drinking PAW also share positive health benefits. In addition, preliminary data indicate positive effects of drinking PAW on mice with fat disease and with diabetes. Detailed mechanisms related to these attractive findings based on PAW in medicine are further investigated in process. The non-toxic PAW possesses high potential in clinical medicine associated with inflammatory-related diseases.

## Figures and Tables

**Table 1:** The published functional water in the references.

Different Kinds of Water	Preparation Methods	Novel Properties	Representative Reference
Hydrogen-rich water	Adding saturated hydrogen gas in water	Scavenging free radicals	[13]
Acidic cosmetic water	Adding acidic compound in water	Scavenging free radicals	[19]
Sulfurous water	Adding sulfur compound in water	Scavenging free radicals	[20]
Engineered water	Electrospraying water vapor	Inactivate foodborne microorganisms	[42]
Magnetic water	Applying magnetic fields on water	Reduced hydrogen bonds (HBs)	[45]
Plasmon-activated water/this work	Resonant illumination on gold-supported nanoparticles (AuNPs) in water	Scavenging free radicals, reduced HBs and energetic	[23]
